# Hepatitis C Virus in the Hematology/Oncology Patient

**Published:** 2017-11-01

**Authors:** Wendy H. Vogel

Hepatitis C virus (HCV) is an acute viral infection of the liver, with symptoms that may include fever, headache, malaise, anorexia, nausea, vomiting, diarrhea, and abdominal pain and either jaundice or elevated serum alanine aminotransferase (ALT) levels of > 400 IU/L (Centers for Disease Prevention and Control [CDC], 2015). Hepatitis C accounts for more deaths in the United States than does hepatitis A and B (CDC, 2015).

The incidence rates of hepatitis C infection are increasing in the United States. There were more than 29,700 estimated new cases in the United States in 2013 (CDC, 2015) compared with 16,500 in 2011—a 55% increase. A total of 75% of patients who have chronic HCV infection were born between 1945 and 1965. The incidence rate of acute HCV in patients aged 20 to 29 is higher than in any other age group, followed by those between the ages of 30 and 39. This rate has increased precipitously since 2010. The incidence rate is slightly higher in males and in American Indian/Alaskan Natives, followed by whites and non-Hispanics (CDC, 2015).

## RISK FACTORS

Risk factors for HCV infection include injecting illicit drugs; a male having sex with men; sexual contact; multiple sexual partners; dialysis or kidney transplant; needle sticks; surgery; and certain occupations such as those in the medical or dental field or other fields that involve contact with human blood (CDC, 2015). The most common risk factors for HCV infection are drug injection; history of blood transfusion prior to 1992; or the presence of abnormal serum ALT level (CDC, 2015; Terrault & Chopra, 2017). Table 1 lists populations that should be tested for HCV. Unlike hepatitis A and B, there is no available vaccine to prevent HCV.

**Table 1:**
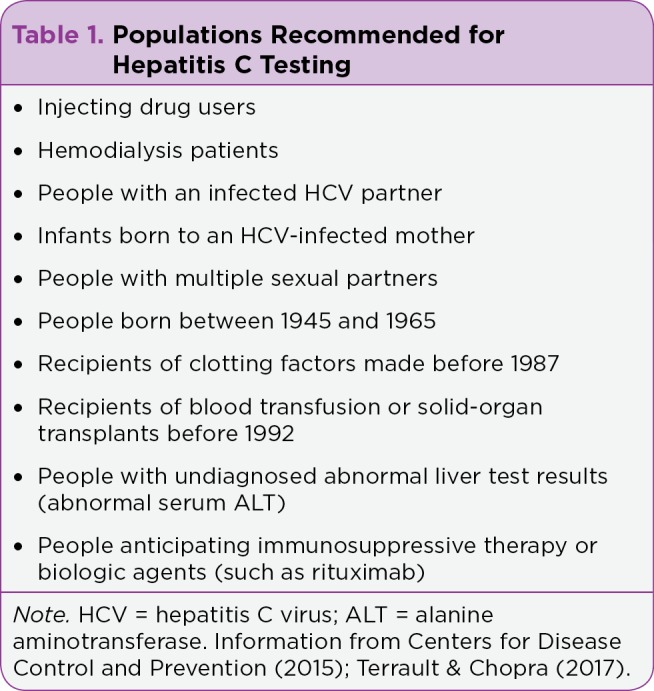
Populations Recommended for Hepatitis C Testing

## CLINICAL PRESENTATION

Acute HCV infection is defined as the first 6 months of infection after exposure. Most patients with acute HCV infection are not jaundiced and may have few, if any, symptoms. Most patients go undetected (Lorenz & Endres, 2017). Symptoms may appear within 2 to 26 weeks after exposure and might include jaundice, nausea, dark urine, and right upper quadrant pain. Patients with acute HCV infection usually have elevated serum aminotransferase levels.

If acute infection is diagnosed, serial monitoring of the HCV RNA is recommended to determine whether the virus is spontaneously cleared or chronic infection occurs. A sustained clearance is defined by at least two negative HCV RNA tests that are at least 12 weeks apart (Lorenz & Endres, 2017).

It is sometimes difficult to determine whether the HCV infection is acute or chronic. If the patient has a positive HCV RNA test (polymerase chain reaction analysis for RNA quantification) and there are no detectable anti-HCV antibodies, then this is considered diagnostic of acute infection. It is also likely to be an acute infection if there were negative tests within the prior 6 months and current tests are positive (Lorenz & Endres, 2017). Repeating HCV RNA testing about 6 weeks after the first positive test may help to diagnose chronic infection. Higher HCV levels that do not fluctuate are indicative of chronic infection. In acute HCV infection, there are lower HCV levels that fluctuate.

Most untreated patients will develop chronic HCV infection. Some patients will go on to develop cirrhosis (about 5%–25%), and some will develop hepatocellular carcinoma (Bojito-Marrero & Pyrsopoulos, 2014). The risk for an earlier development of cirrhosis and fibrosis is increased in patients who have undergone hematopoietic stem cell transplant (Borchardt & Torres, 2014).

## TREATMENT SELECTION BY GENOTYPE

There are several different genotypes of HCV, and they may vary according to geographic location (Borchardt & Torres, 2014). In the United States, genotype 1 is the most common, followed by genotypes 2 and 3. Genotype 1 is also the most prevalent in cancer patients, yet genotype 2 is the most common genotype in lymphoma patients (Borchardt & Torres, 2014). Treatment selection, duration, and dosing may vary according to genotype.

Hepatitis C guidelines recommend treatment consideration in all patients with a life expectancy of more than 12 months (Chopra & Arora, 2017). Obtaining a sustained viral response is desired and can reduce morbidity, even if the patient has advanced fibrosis/cirrhosis (Chopra & Arora, 2017). Treatment decisions are based on genotype, the existence of viral resistance, a history of treatment, fibrotic stage, comorbid conditions, concurrent drug/alcohol use, psychiatric history, and any underlying autoimmune disorders.

## HEPATITIS C IN THE ONCOLOGY/HEMATOLOGY PATIENT

However, there is little information on the impact of HCV in the oncology or hematology patient. Although chronic HCV infection is not unusual in these patients, there are few studies on the unique challenges of this patient population (Borchardt & Torres, 2014). There have been rare cases of acute exacerbation and reactivation of HCV in patients receiving biologic therapy such as rituximab (Rituxan; Bojito-Marrero & Pyrsopoulos, 2014; Mahale et al., 2012; Paydas, 2014; Sagnelli, Pisaturo, Sagelli, & Coppola, 2012). Mahale and colleagues (2012) reported that 36% of patients with known HCV had viral reactivation following chemotherapy (n = 22). Acute exacerbation of HCV occurred in 11% of patients (n = 308). The populations most likely to have acute exacerbation have hematologic malignancies and lymphopenia.

Several biologic agents have been associated with acute exacerbation/reactivation of HCV, including rituximab, alemtuzumab (Campath), and tumor necrosis factor (Bojito-Marrero & Pyrsopoulos, 2014; Mahale et al., 2012). Chemotherapy was more likely to be discontinued in patients with acute exacerbation of HCV due to liver dysfunction.

Most patients with HCV may receive cancer treatment safely. Patients should be monitored for febrile neutropenia (Miura et al., 2013) and elevations in liver transaminase levels (Morrow et al., 2009). It is prudent to consult with gastroenterology in patients with HCV prior to chemotherapy administration to assist in the determination of risk secondary to the chemotherapy. Treatment of the cancer generally takes precedence over treatment of HCV. A possible exception to this rule might be the patient with HCV and hepatocellular cancer, and there is a need to treat the HCV prior to liver transplantation (Tutt, 2013).

However, the hepatotoxicity of many oncologic agents may be detrimental to the patient with cirrhosis, and some HCV treatments may be immunosuppressive. For instance, interferon-based therapy may be immunosuppressive, whereas direct-acting antiviral (DAA) regimens are usually not but could cause anemia. However, these DAA regimens may not be an option due to access issues or financial reasons (Lorenz & Endres, 2017).

Two case studies of young adult oncology/hematology patients who were diagnosed with HCV follow. 

## CASE STUDY 1: HEPATITIS C IN A PATIENT WITH TREATED HODGKIN LYMPHOMA

Patient ZM was diagnosed with Hodgkin lymphoma, stage IIIB in September 2013. He reported a history of marijuana use and alcohol abuse and denied other illicit drug use. He self-reported a negative hepatitis screen in 2013. He was treated with ABVD (doxorubicin, bleomycin, vinblastine, dacarbazine) × 6 cycles for his cancer, completing the treatment on March 2014. He has been in complete remission since that time and was under active observation at the time of his HCV diagnosis. 

In August 2014, 5 months after completion of the treatment, he presented to the emergency department (ED) for edema in his neck and was fearful his cancer had returned. He also had a sore throat. He was placed on cephalexin, and computed tomography (CT) was ordered. Computed tomography of the neck and chest showed no recurrence.

When seen by oncology following the ED visit, his bilirubin was elevated to 1.4 mg/dL, aspartate aminotransferase (AST) was 85 U/L, and ALT was 156 U/L. An acute hepatitis panel was ordered as well as an Epstein-Barr virus (EBV) antibody panel. Epstein-Barr virus immunoglobulin G (IgG) was elevated at > 750 U/mL, indicating a previous EBV infection. He had a reactive hepatitis C antibody in August 2014. 

Computed tomography scans of the abdomen and pelvis were performed in September 2014, showing no evidence of Hodgkin lymphoma, hepatomegaly, or splenomegaly. He admitted to illicit drug use through snorting. He has tattoos but has had no blood transfusions and no jail time. He admitted to unprotected sex. He was referred to gastroenterology.

Gastroenterology ordered hepatitis C RNA quantitative and hepatitis C genotype to assess the viral load and genotype. However, the patient did not have any insurance and did not show up for this blood work or for further gastrointestinal office visits. He also did not return for any follow-up visits to oncology after September 2014, despite numerous attempts to reach him.

This patient has risk factors for HCV. However, since he did not undergo further testing, it is unknown whether it was an acute HCV infection or not. As with many young cancer survivors, he exhibited risky behaviors, such as continued smoking, unprotected sex, and illicit drug use. Noncompliance with follow-up in this population is also common.

## CASE STUDY 2: HEPATITIS C IN A HEMATOLOGY PATIENT

Patient TN has a history of immune thrombocytopenic purpura (ITP), diagnosed at age 12 in 2006, and is followed by pediatric hematology oncologists. He was previously treated with prednisone and intravenous immunoglobulin in 2012. A bone marrow biopsy and aspiration in July 2014 was unremarkable, with an adequate number of megakaryocytes.

He presented to hematology in 2012 at age 19 seeking medical clearance for joining the Navy. The platelet count at this time was 96,000/∝L. He did not keep scheduled follow-up appointments. In April 2013, he presented to the primary care office due to headaches, and the platelet count was found to be 15,000/∝L. He was referred back to hematology, and the platelet count was noted to be 37,000/∝L in April 2013. He was treated with dexamethasone, and his platelet count recovered. He was then lost to follow-up.

In July 2014, a phone call from TN’s mother noted he had blood in his stool and an episode of hematemesis. The platelet count had dropped to 22,000/∝L later in July, and hemoglobin level was 8.2 g/dL, hematocrit 25.6%. A hematology workup ensued (comprehensive metabolic panel, bilirubin, direct antiglobulin test, ferritin, folate, haptoglobin, iron and total iron-binding capacity, lactate dehydrogenase [LDH], retic, vitamin B12). Vitamin B12, folate, and iron were replete. Iron saturation was 58%, and ferritin was 256 ng/mL. The direct antiglobulin test was negative. He had no signs of bleeding or symptoms of anemia and was placed on prednisone. The platelet count increased to 64,000/∝L by the end of July.

At his next clinic visit, early in August, his platelet count was noted to be 30,000/∝L, and track marks were noted in his left antecubital area, although he denied any illicit drug use with the exception of marijuana. A drug screen was ordered, as well as a heavy metal screen, urine bath salt screen, urine synthetic cannabinoid screen, and hepatitis panel.

His hepatitis C antibody was reactive, and LDH was elevated at 615 U/L early in August. He was anemic, with a hemoglobin of 8.0 g/dL and hematocrit of 24.8%. The platelet count was 30,000/∝L. The heavy metals panel was negative, and the drug screen was positive for oxymorphone (a metabolite of oxycodone) and cannabinoids.

On a follow-up in the middle of August, he admitted to illicit drug use, including intravenous use of oxymorphone. Due to deteriorating blood cell counts and his history of abuse, acute hepatitis panel, HIV testing, bone marrow aspiration, and biopsy and CT scans of the chest, abdomen, and pelvis were ordered. The HCV RNA quantitative was high at 625,826 IU/mL, and RNA log was 5.80 log 10. He was HIV-negative. He was referred to gastroenterology.

Bone marrow aspiration and biopsy were performed late in August 2014, which showed normocellular bone marrow with an adequate number of megakaryocytes and no evidence of malignancy. Severe thrombocytopenia was noted as well as moderate, marginally macrocytic, normochromic anemia. The report noted peripheral blood and bone marrow findings consistent with thrombocytopenia that is related to increased peripheral destruction and/or splenic sequestration and compatible with the clinical diagnosis of ITP. Computed tomography scans of the abdomen and pelvis demonstrated no hepatomegaly, and the spleen size was in the upper limit of normal.

The patient was seen by gastroenterology in September. Hepatitis C genotype, antinuclear antibodies, and hepatitis A and B antibodies tests were ordered. Hepatitis A antibody was positive, hepatitis B antibody was negative, and hepatitis C virus genotype was 2. The antinuclear antibodies test was negative. Interferon treatment was considered contraindicated due to thrombocytopenia and anemia. Recommendations from gastroenterology for hepatitis C genotype 2 were sofosbuvir plus ribavirin, but due to severe thrombocytopenia, the risks of treatment were considered high. He was referred to a university tertiary center for treatment recommendations for both hematology and hepatology.

Throughout his treatment with hematology and gastroenterology, TN was noncompliant with requested lab work, steroid intake, and scheduled clinic visits. He was insured through his parents’ insurance. However, he did not want any information about his disease status shared with them. He got married during this time, and soon after, his wife also tested positive for HCV. Periodically throughout care with hematology, he would admit to intravenous use of buprenorphine and naloxone, and subsequently the platelet count would drop precipitously.

He was seen by the tertiary center, who noted he was steroid-dependent. Treatment of his ITP is complicated: rituximab is contraindicated because of HCV. Splenectomy was recommended with liver biopsy concurrently to stage liver disease. Hepatology concurred, noting he likely has early-stage disease with no evidence of cirrhosis. The plan for hepatitis treatment would ensue once the platelet count recovered or when he required lower doses of steroids. The plan of treatment included daclatasvir and sofosbuvir for 12 weeks.

He underwent splenectomy in January 2016, and a liver biopsy was performed at the same time. He was found to have an accessory spleen. Liver biopsy showed minimal portal inflammation with minimal portal expansion and no significant fibrosis.

As in the first case study, caring for this young adult patient was challenging due to his noncompliance and risky behaviors. Even if successful sustained virologic response occurs with daclatasvir and sofosbuvir, the continued risky behaviors place him at risk for reinfection. Collaboration with hepatology provided guidance for treatment in this complicated situation.

## DIAGNOSTIC TESTING

There are some challenges in diagnosing chronic HCV in the oncology/hematology patient (Borchardt & Torres, 2014). An HCV antibody test is the initial screening test of choice (Terrault & Chopra, 2017). Patients with HCV should be tested for HIV and both hepatitis A and B as well. If the HCV antibody test is reactive, then an HCV RNA test (polymerase chain reaction analysis for RNA quantification) should be ordered. If the patient is immunocompromised or has a hematologic malignancy, the HCV antibody test may be falsely negative. False-negative results are more common and will still require further workup for the diagnosis with the HCV RNA test (Borchardt & Torres, 2014). If acute HCV infection is suspected, immediate and sequential HCV RNA and liver enzyme testing is recommended.

## MANAGEMENT

The goal of HCV treatment is a sustained virologic response (absence of virus in the blood 12 weeks after treatment cessation; Chopra & Arora, 2017). General resources for the diagnosis and treatment of HCV are noted in Table 2. There is no standard treatment or guidelines for the treatment of HCV in the oncology/hematology population. Clinical trials in HCV often exclude patients with cancer.

**Table 2:**
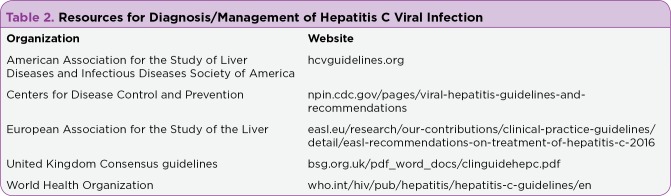
Resources for Diagnosis/Management of Hepatitis C Viral Infection

The goal of medical management is to eradicate HCV RNA primarily with antiviral therapy. Obtaining a sustained virologic response is associated with a 97% to 100% chance of being HCV RNA–negative and may be considered a cure. Most HCV treatment guidelines opt for not treating acute HCV but observing patients for spontaneous clearance for at least 6 months. Initiation of treatment would then ensue when chronic disease occurs (Lorenz & Endres, 2017). Patients with a detectable HCV viral level over 6 months (chronic HCV) should be considered for treatment. The treatment regimen and duration will vary according to genotype and patient factors (Lorenz & Endres, 2017). Interferon is often used in the acute setting and is quite efficacious. Direct-acting antiviral agents have not been as well studied in the acute setting and are often cost-prohibitive, as insurers often require documentation of HCV infection for 6 months prior to DAA initiation (Lorenz & Endres, 2017).

Treatment of HCV concomitantly with chemotherapy must be considered carefully, although there is no consensus about the management of HCV during chemotherapy or immunosuppressive states (Hwang & Liang, 2010; Paydas, 2014; Tomizawa et al., 2014). Even in those who are not treated concomitantly, HCV treatment could potentially cause more toxicity in the patient with cancer (Borchardt & Torres, 2014).

Initial baseline hematologic values may be compromised from prior cancer treatment, leading to rapid and deeper hematologic nadirs from HCV therapy such as interferon. Borchardt and Torres (2014) recommend waiting at least 6 months after cancer remission to begin HCV treatment if utilizing interferon and ribavirin, avoiding concomitant HCV therapy with chemotherapy. Unlike interferon regimens, treatment with DAA agents are generally well tolerated and have few contraindications (Chopra & Arora, 2017). However, drug-drug interactions are a concern, particularly with protease inhibitors (such as grazoprevir, paritaprevir, and simeprevir; Pockros, 2017).

Anemia may also occur with protease inhibitors, as can rash and photosensitivity. Simeprevir is not recommended in those with moderate to severe hepatic impairment. Anemia may occur with sofosbuvir. Other common side effects of the DAA agents are fatigue, headache, nausea, insomnia, and renal impairment (Borchardt & Torres, 2014; Pockros, 2017).

In a patient with known HCV infection who is undergoing biotherapy and/or chemotherapy, the liver function and HCV viral load should be monitored carefully, particularly when there are abnormal liver function tests prior to treatment initiation (Paydas, 2014). Cancer treatment modification may be required, according to the prescribing information of the cancer treatment. It is not clear whether antiviral prophylaxis will suppress HCV while a patient is receiving cancer treatment. A positive HCV serology does not preclude cancer treatment (Tomizawa et al., 2014).

Fatigue is common in HCV and in the hematology/oncology patient. Almost all treatments for HCV, especially interferon, can compound fatigue. Chemotherapy agents can be hepatotoxic, and care should be taken when administering these treatments to patients with chronic hepatitis.

Response to treatment is assessed by rechecking the viral load. In acute and chronic HCV infection, viral response to treatment is recommended at about 12 weeks after cessation of treatment (Lorenz & Endres, 2017). No detection of the virus is considered a virologic response.

## PATIENT EDUCATION

Patients should be educated about obesity management, tobacco cessation (if applicable), and marijuana smoking cessation, as all of them may increase the risk of hepatic fibrosis and ultimately increase the risk for hepatocellular cancer (Borchardt & Torres, 2014). Diabetes should be managed closely. Avoidance of hepatotoxic agents such as alcohol and acetaminophen is recommended (Lorenz & Endres, 2017). Patients should be counseled about high-risk behaviors that increase the risk of reinfection or transmission. The risk for hepatocellular carcinoma occurs at a rate of 1% to 4 % per year in patients with cirrhosis. Patients with HCV have an increased risk for marginal zone lymphoma, diffuse large B-cell lymphoma, and lymphoplasmacytic lymphoma (Nooka, Shenoy, Sinha, Lonial, & Flowers, 2011). 

## PSYCHOSOCIAL MANAGEMENT

Patients diagnosed with HCV have common concerns about sexual transmission and risk of infecting other persons (Marinho & Barreira, 2013). Depression is common in both the oncology patient and the patient with HCV. Uncontrolled depression may be a contraindication for HCV treatment with interferon (Borchardt & Torres, 2014). There are unique concerns for the oncology/hematology patient diagnosed with HCV, including the effects on current treatment, additional side effects for HCV treatment, and risks of future cancers. The diagnosis of HCV has a stigma associated with it that can influence depression, anxiety, fatigue, and other mental health problems (Marinho & Barreira, 2013). This stigma can lead to feelings of isolation and poor self-esteem and may impact relationship intimacy. Patients and significant others may blame the patient with HCV, as risky behaviors are often the cause of HCV.

## UNIQUE CHALLENGES OF ONCOLOGY/HEMATOLOGY PATIENTS AND YOUNG ADULTS

It is important to assess the oncology/hematology patient at any age for a history of HCV. Hepatitis C reactivation can occur during chemotherapy or immunotherapy (such as rituximab). The diagnosis of hepatitis may impact the choice of cancer therapy. Cancer therapy may need to be interrupted (Nooka et al., 2011). Alternatively, the treatment for HCV may need to be delayed. The potential for drug-drug interaction between the cancer and HCV agents exists, especially with DAA agents. Patients with cancer may have a compromised response to HCV treatment (Borchardt & Torres, 2014).

A young patient (late adolescent or young adult between the ages of 16 and 39) may have additional psychosocial issues that complicate oncologic or HCV treatments. As in both of the case studies presented, nonadherence and risky behaviors can be a concern. Young adults and adolescents with cancer may be more likely to engage in risky behaviors (Murphy, Klosky, Termuhlen, Sawczyn, & Quinn, 2013; Pilapil & DeLaet, 2015; Ruiz, Sender, Torno, & Fortier, 2016). Nonadherence can adversely affect clinical outcomes in both hepatitis and cancer.

In patients with chronic hematologic disorders, nonadherence is common in up to 50% of patients and actually increases with age (Leader & Raanani, 2014). The National Cancer Institute notes that adolescents and young adults (aged 16–39) are a vulnerable population, as improvements in survival have not occurred in the younger and older populations (Bleyer, 2007; Wood & Lee, 2011). This age group also has other challenges such as lack of insurance, access to care, delays in diagnosis, inadequate representation in clinical trials, and psychosocial issues common to this age (such as growing independence, parents of young children, and certain supportive care needs, as well as social concerns including peer relationships, romantic relationships, and financial security; Ruiz et al., 2016; Wood & Lee, 2011).

Recognizing these unique challenges, the American Society of Clinical Oncology (ASCO), the Association of Physician Assistants in Oncology (APAO), and several other organizations have partnered together to offer educational initiatives for this age group, called "Focus Under Forty" (ASCO, 2017).

## CONCLUSION

The oncology advanced practitioner must be knowledgeable about HCV, risk factors, and the diagnosis and management of HCV. Knowing when to refer a patient to a specialist during the cancer trajectory is vital. Patients with cancer and HCV face additional challenges that require education, support, and medical vigilance on the part of the oncology advanced practitioner. The adolescent or young adult with these diagnoses may present with additional issues that could impact morbidity and mortality.
